# Relative Effectiveness of High-Dose Versus Standard-Dose Influenza Vaccination Against Hospitalizations and Deaths According to Frailty Score: A Post Hoc Analysis of the DANFLU-1 Randomized Trial

**DOI:** 10.1093/infdis/jiaf420

**Published:** 2025-08-13

**Authors:** Caroline Espersen, Niklas Dyrby Johansen, Daniel Modin, Kira Hyldekær Janstrup, Joshua Nealon, Sandrine Samson, Matthew M Loiacono, Rebecca C Harris, Melissa K Andrew, Carsten Schade Larsen, Anne Marie Reimer Jensen, Nino Emanuel Landler, Brian L Claggett, Scott D Solomon, Martin J Landray, Gunnar H Gislason, Lars Køber, Jens Ulrik Stæhr Jensen, Pradeesh Sivapalan, Tor Biering-Sørensen

**Affiliations:** Department of Cardiology, Copenhagen University Hospital–Herlev and Gentofte, Copenhagen, Denmark; Center for Translational Cardiology and Pragmatic Randomized Trials, Department of Biomedical Sciences, Faculty of Health and Medical Sciences, University of Copenhagen, Copenhagen, Denmark; Department of Cardiology, Copenhagen University Hospital–Herlev and Gentofte, Copenhagen, Denmark; Center for Translational Cardiology and Pragmatic Randomized Trials, Department of Biomedical Sciences, Faculty of Health and Medical Sciences, University of Copenhagen, Copenhagen, Denmark; Department of Cardiology, Copenhagen University Hospital–Herlev and Gentofte, Copenhagen, Denmark; Center for Translational Cardiology and Pragmatic Randomized Trials, Department of Biomedical Sciences, Faculty of Health and Medical Sciences, University of Copenhagen, Copenhagen, Denmark; Department of Cardiology, Copenhagen University Hospital–Herlev and Gentofte, Copenhagen, Denmark; Center for Translational Cardiology and Pragmatic Randomized Trials, Department of Biomedical Sciences, Faculty of Health and Medical Sciences, University of Copenhagen, Copenhagen, Denmark; Sanofi, Lyon, France; Sanofi, Lyon, France; Sanofi, Pennsylvania, USA; Sanofi, Singapore; Geriatric Medicine Research, Department of Medicine, Dalhousie University, Halifax, Nova Scotia, Canada; Department of Clinical Medicine, Department of Infectious Diseases, Aarhus University Hospital, Aarhus, Denmark; Department of Cardiology, Copenhagen University Hospital–Herlev and Gentofte, Copenhagen, Denmark; Center for Translational Cardiology and Pragmatic Randomized Trials, Department of Biomedical Sciences, Faculty of Health and Medical Sciences, University of Copenhagen, Copenhagen, Denmark; Department of Cardiology, Copenhagen University Hospital–Herlev and Gentofte, Copenhagen, Denmark; Center for Translational Cardiology and Pragmatic Randomized Trials, Department of Biomedical Sciences, Faculty of Health and Medical Sciences, University of Copenhagen, Copenhagen, Denmark; Cardiovascular Division, Brigham and Women's Hospital, Harvard Medical School, Boston, Massachusetts, USA; Cardiovascular Division, Brigham and Women's Hospital, Harvard Medical School, Boston, Massachusetts, USA; Nuffield Department of Public Health, University of Oxford, Oxford, United Kingdom; Department of Cardiology, Copenhagen University Hospital–Herlev and Gentofte, Copenhagen, Denmark; Department of Clinical Medicine, Faculty of Health and Medical Sciences, University of Copenhagen, Copenhagen, Denmark; The Danish Heart Foundation, Copenhagen, Denmark; The National Institute of Public Health, University of Southern Denmark, Copenhagen, Denmark; Department of Cardiology, Copenhagen University Hospital—Rigshospitalet, Copenhagen, Denmark; Department of Clinical Medicine, Faculty of Health and Medical Sciences, University of Copenhagen, Copenhagen, Denmark; Respiratory Medicine Section, Department of Medicine, Copenhagen University Hospital–Herlev and Gentofte, Copenhagen, Denmark; Department of Clinical Medicine, Faculty of Health and Medical Sciences, University of Copenhagen, Copenhagen, Denmark; Respiratory Medicine Section, Department of Medicine, Copenhagen University Hospital–Herlev and Gentofte, Copenhagen, Denmark; Department of Cardiology, Copenhagen University Hospital–Herlev and Gentofte, Copenhagen, Denmark; Center for Translational Cardiology and Pragmatic Randomized Trials, Department of Biomedical Sciences, Faculty of Health and Medical Sciences, University of Copenhagen, Copenhagen, Denmark; Department of Cardiology, Copenhagen University Hospital—Rigshospitalet, Copenhagen, Denmark

**Keywords:** influenza, vaccine, frailty, pragmatic, randomized controlled trial

## Abstract

**Background:**

Frailty is a risk factor for adverse influenza-related outcomes. We assessed the effectiveness of high-dose inactivated influenza vaccination (HD-IIV) versus standard-dose inactivated influenza vaccination (SD-IIV) according to a frailty score.

**Methods:**

This was a post hoc analysis of the randomized feasibility trial of HD-IIV versus SD-IIV conducted during the 2021–2022 influenza season in older adults aged 65–79 years. We assessed prespecified outcomes, including hospitalizations and deaths, as first and recurrent events. Frailty was defined according to the hospital frailty risk score (HFRS).

**Results:**

Among 12 477 included participants (mean age, 71.7 years; 47.1% female), 10 689 (85.7%) were categorized as having low frailty (HFRS <5) and 1784 (14.3%) had intermediate or high frailty (HFRS ≥5). HD-IIV versus SD-IIV was associated with a lower risk of first and recurrent hospitalizations for pneumonia or influenza regardless of the HFRS. For participants with low frailty there were 22 events (hazard ratio [HR], 0.37 [95% confidence interval, .15–.96]) and 25 recurrent events (incidence rate ratio, 0.31 [.11–.84]), and for those with intermediate or high frailty there were 16 events (HR, 0.33 [.11–1.01]) and 18 recurrent events (incidence rate ratio, 0.28 [.09–.92]; *P* value for continuous interaction with HFRS [*P*_interaction_] = .92 for first and *P*_interaction_ = .93 for recurrent events). The HFRS modified the association of HD-IIV versus SD-IIV with all-cause mortality (*P*_interaction_ = .02), with an association with reduced risk in participants with low frailty only (43 events, HR, 0.26 [95% confidence interval, .13–.55]).

**Conclusions:**

HD-IIV was associated with a lower risk of first and recurrent hospitalizations for pneumonia and influenza compared with SD-IIV and may be preferred for older adults, irrespective of frailty status. The HFRS modified the association of HD-IIV versus SD-IIV with all-cause mortality.

## Teaser

In a post hoc DANFLU-1-analysis, high-dose versus standard-dose influenza vaccination reduced hospitalizations for pneumonia/influenza irrespective of frailty. The frailty score modified the effect of high-dose influenza vaccination on all-cause mortality, with evidence of effect only in low-frailty participants.

With an estimated 1 billion cases annually worldwide according to the World Health Organization [1], seasonal influenza represents a great burden for society and the healthcare system especially among the older, frail population. Older adults are at higher risk of adverse outcomes on contracting influenza, with higher hospitalization and mortality rates [2–6] attributable to suboptimal immune responses in older adults [3], the multiplicative impacts of frailty [7], and the elevated prevalence of comorbid conditions in this population.

Influenza vaccination is recommended for all individuals ≥65 years of age in Europe and more broadly in the United States [8, 9], and it is effective in preventing influenza infection and influenza-related hospitalizations and deaths [10–12]. More recently, influenza vaccination has also been shown to protect against nonspecific hospitalizations and deaths, including fatal and nonfatal cardiovascular events [13–16]. However, due to “immunosenescence,” immune responsiveness to vaccination has been shown to be reduced in older individuals [17]. In addition, frailty may contribute to lower vaccine efficacy and effectiveness [18–20].

High-dose influenza vaccination (HD-IIV) was developed to improve protection against influenza and influenza-related complications among older adults. In large, randomized trials, HD-IIV has been shown to improve vaccine immunogenicity and efficacy compared with standard-dose influenza vaccination (SD-IIV) in older adults [21, 22] and could represent a promising approach to lower the risk of influenza-related complications particularly among the high-risk older, frail population. Nonetheless, data are sparse regarding the added benefit of HD-IIV versus SD-IIV on the risks of specific and nonspecific hospitalizations and deaths according to frailty status. Therefore, we sought to explore signals indicating potential differences in the relative effectiveness of HD-IIV versus SD-IIV against first and recurrent hospitalizations and deaths according to frailty status in older individuals. Moreover, we sought to investigate the difference in frailty scores from vaccination to the end of follow-up in individuals receiving HD-IIV versus SD-IIV.

## METHODS

### Study Design

This was a post hoc analysis of the DANFLU-1 trial. The study design and primary findings of the DANFLU-1 trial have been published elsewhere [23, 24]. In brief, the DANFLU-1 trial was a pragmatic, registry-based, open-label, active-controlled, randomized feasibility trial investigating HD-IIV versus SD-IIV in older adults during the 2021–2022 northern hemisphere influenza season. The purpose of the trial was to evaluate the feasibility of conducting large-scale pragmatic vaccine trials within the Danish healthcare system, using nationwide registries for baseline, safety, and end point assessments [23]. The Regional Danish Committee on Biomedical Research Ethics (no. H-21035316) and the Danish Medicines Agency (EudraCT no. 2021-003170-31) approved the study. The trial is registered at Clinicaltrials.gov (NCT05048589).

The eligibility criteria for participation in the trial were (1) age 65–79 years at enrollment and (2) no study vaccine allergies. A private vaccination provider responsible for organizing vaccination appointments under the Danish governmental vaccination program recruited participants for the trial. A central site monitored the study throughout the study period and was responsible for registry-based data collection and safety monitoring. Participants were enrolled in the trial from 1 October to 20 November 2021, and all provided written informed consent before participation.

### Randomization

Participants were randomized in a 1:1 ratio to HD-IIV or SD-IIV using central blocked randomization. This was an open-label study; investigators, participants, and study personnel were not blinded to treatment assignment. However, as all subsequent data were retrieved passively from nationwide registries containing routinely obtained health data, the risk of ascertainment bias was minimized.

### Study Treatment and Clinical Information

Vaccines for HD-IIV (Fluzone High-Dose Quadrivalent [United States and Canada]/Efluelda [Europe]; Sanofi) contained 60 mg of hemagglutinin antigen for each influenza strain, compared with 15 mg for each with SD-IIV. Both vaccine types contained the same 4 influenza strains as recommended by the World Health Organization for the 2021–2022 northern hemisphere influenza season. All vaccines administered for SD-IIV were InfluvacTetra (Viatris).

Personal identifying information and information on the randomization group were collected by the vaccination providers and subsequently transferred to the central site. All other trial data were obtained from the nationwide health registries by the central site investigators by linking the registry data to each participant using a unique social security number. The Danish nationwide registries contain data on all hospital contacts (both inpatient and outpatient contacts), procedures, and redeemed prescriptions in the Danish public healthcare system [24, 26].

Information on baseline diagnoses, medication use, and outcomes were obtained using prespecified definitions based on the *International Classification of Disease, Tenth Revision* (*ICD-10*) and Anatomical Therapeutic Chemical classification codes, as described elsewhere [23, 24]. Baseline diagnoses were assessed within 10 years before the enrollment date.

### Frailty Score Calculation

The frailty risk was calculated according to the hospital frailty risk score (HFRS) [27], based on *ICD-10* codes for relevant comorbid conditions present at any time before vaccination and using the Danish nationwide registries, including both outpatient and inpatient visits. In brief, by assigning weighted points to a list of relevant comorbid conditions and adding the points for all conditions present, the HFRS represents the overall frailty risk for each individual. The higher the HFRS, the higher the frailty risk. Participants were divided into 2 groups according to the HFRS; low frailty (HFRS <5) and intermediate or high frailty (HFRS ≥5) [27]. We also calculated the HFRS at the end of follow-up, using the same definition. Supplementary Table 1 lists the calculation of the HFRS with the individual *ICD-10* codes and the prevalence of each *ICD-10* code in the DANFLU-1 study population at baseline.

### Outcomes

We assessed the following prespecified outcomes in relation to frailty status: hospitalizations for (1) pneumonia or influenza, (2) respiratory disease, (3) cardiorespiratory disease, (4) cardiovascular disease, (5) all causes and (6) all-cause mortality rate. We evaluated both first and recurrent events for all outcomes other than all-cause mortality, which was evaluated as time to first event only. To assess the total burden of patient-relevant outcomes, we chose to analyze recurrent events. Analyzing recurrent events may be beneficial in single-administration vaccine trials, as the risk of treatment discontinuation after a first event is eliminated when the intervention is administered once at baseline [28]. The follow-up period for clinical outcomes spanned from 14 days after vaccination until 31 May 2022.

### Statistical Analysis

All analyses were performed according to the intention-to-treat principle. As DANFLU-1 was a pilot trial, it was not powered for clinical outcomes. These analyses should therefore be considered exploratory and hypothesis generating only. Baseline characteristics are stratified by frailty status and summarized with mean (standard deviation [SD]) or median (interquartile range [IQR]) for normally or nonnormally distributed continuous variables, respectively, and with absolute numbers and percentages for categorical variables. Continuous variables are compared using Student *t* or Wilcoxon rank sum tests as appropriate. Categorical variables are compared using χ^2^ or Fisher exact tests as appropriate. For time-to-first-event outcomes, we used Cox regression to calculate hazard ratios (HRs) comparing HD-IIV versus SD-IIV among patients with low and intermediate or high frailty. Restricted cubic spline plots constructed using the STATA mkspline and Poisson commands were used to depict incidence rate ratio (IRRs) comparing the association of HD-IIV versus SD-IIV with all-cause mortality rate according to the HFRS. The number of knots was chosen based on the lowest Akaike information criterion.

For recurrent events, we used negative binomial regression to calculate IRRs comparing HD-IIV versus SD-IIV in participants with low and intermediate or high frailty. Follow-up time was included as an offset in the Poisson and negative binomial regression models. Recurrent readmissions were counted regardless of the time between admissions. As such, all hospital readmissions occurring in the follow-up period were counted. To further account for any associations between recurrent events, we also performed an Andersen-Gill Cox regression analysis for recurrent events. We also evaluated the interaction between the HFRS as a continuous variable and HD-IIV versus SD-IIV and the prespecified outcomes in both Cox and negative binomial regression analyses. For both Cox and negative binomial regression, 95% confidence intervals (CIs) were calculated using asymptotic Wald intervals. Hospitalization rates per 100 person-years in patients with low and intermediate or high frailty were compared using unadjusted negative binomial regression models. Finally, we compared the HFRS at end of follow-up and the increase in the HFRS from vaccination to end of follow-up in participants receiving HD-IIV versus SD-IIV, using both Student *t* and Wilcoxon rank sum tests. The change in the HFRS from vaccination to the end of follow-up for each individual was compared using Wilcoxon signed rank test. Participants who died during follow-up were excluded from this analysis. All analyses were performed without adjustment for multiple testing. Differences were considered statistically significant at *P* < .05. We used SAS Software, version 9.4 (SAS Institute), Stata MP, version 17.0 (StataCorp), and R, version 4.2.2 (R Foundation for Statistical Computing) for the statistical analysis.

## RESULTS

### Baseline Characteristics According to Frailty Status

Among 12 477 included participants, 6245 were assigned to HD-IIV and 6232 to SD-IIV. Baseline characteristics overall and stratified by randomization group have been published elsewhere [23]. We could not obtain registry data for 4 participants (2 in each randomization group); only information on sex and age was available for these participants. Therefore, we could not calculate the HFRS for these 4 individuals. Overall, the mean age of participants (SD) was 71.7 (3.9) years; 5877 (47.1%) were women, 2540 (20.4%) had cardiovascular disease, and 850 (6.8%) had chronic lung disease (Table 1). The median HFRS (IQR) was 1.10 (0.00–3.30). In total, 10 689 participants (85.7%) were categorized as having low frailty, and 1784 (14.3%) as having moderate or severe frailty. The HFRSs were balanced according to randomization group (median [IQR], 1.1 [0–3.3] for SD-IIV vs 1.1 [0–3.3] for HD-IIV: *P* = .84). Overall, participants with intermediate or high frailty were older (72.5 vs 71.6 years for low frailty) and more likely to have concomitant comorbid conditions, including chronic cardiovascular disease (694 participants [38.9%] vs 1846 [17.3%], respectively), diabetes (253 [14.2%] vs 909 [8.5%]), or chronic lung disease (255 [14.3%] vs 595 [5.6%]) (all *P* < .001) (Table 1).

**Table 1.** Baseline Characteristics According to Frailty Status

Characteristic	Study Participants, No. (%)^a^			*P* Value
	All (N = 12 477)	Low Frailty (n = 10 689)	Intermediate or High Frailty (n = 1784)	
Age, mean (SD), y	71.7 (3.9)	71.6 (3.9)	72.5 (4.0)	<.001
Female sex	5877 (47.1)	5010 (46.9)	866 (48.5)	.19
Chronic lung disease	850 (6.8)	595 (5.6)	255 (14.3)	<.001
COPD	417 (3.3)	267 (2.5)	150 (8.4)	<.001
Chronic cardiovascular disease	2540 (20.4)	1846 (17.3)	694 (38.9)	<.001
Ischemic heart disease	962 (7.7)	700 (6.5)	262 (14.7)	<.001
Heart failure	275 (2.2)	184 (1.7)	91 (5.1)	<.001
Atrial fibrillation	878 (7.0)	667 (6.2)	211 (11.8)	<.001
Hypertension	6469 (51.9)	5285 (49.4)	1184 (66.4)	<.001
Diabetes	1162 (9.3)	909 (8.5)	253 (14.2)	<.001
Cerebrovascular disease	456 (3.7)	204 (1.9)	252 (14.1)	<.001
Cancer	1363 (10.9)	1003 (9.4)	360 (20.2)	<.001
Immunodeficiency	483 (3.9)	354 (3.3)	129 (7.2)	<.001
HFRS, median (IQR)	1.10 (0.00, 3.30)	0.80 (0.00, 2.20)	7.30 (6.00, 9.90)	<.001
HD-IIV	6245 (50.1)	5343 (50.0)	900 (50.4)	.72
Hospitalization in previous influenza season				
Pneumonia or influenza	21 (0.2)	14 (0.1)	7 (0.4)	.02
Respiratory disease	36 (0.3)	23 (0.2)	13 (0.7)	<.001
Cardiovascular disease	166 (1.3)	102 (1.0)	64 (3.6)	<.001
Cardiorespiratory disease	198 (1.6)	123 (1.2)	75 (4.2)	<.001
All causes	992 (8.0)	658 (6.2)	334 (18.7)	<.001

Abbreviations: COPD, chronic obstructive pulmonary disease; HFRS, hospital frailty risk score; HD-IIV, high-dose inactivated influenza vaccination; IQR, interquartile range; SD, standard deviation.

^a^Data represent no. (%) of study participants unless otherwise specified. Low frailty corresponds to a HFRS of <5; intermediate or high frailty, to a HFRS of ≥5. For 4 participants (2 in each randomization group), registry data could not be obtained; only information on sex and age was available for these participants.

### Time-to-First-Event Analysis According to Frailty Status

**Figure 1.**
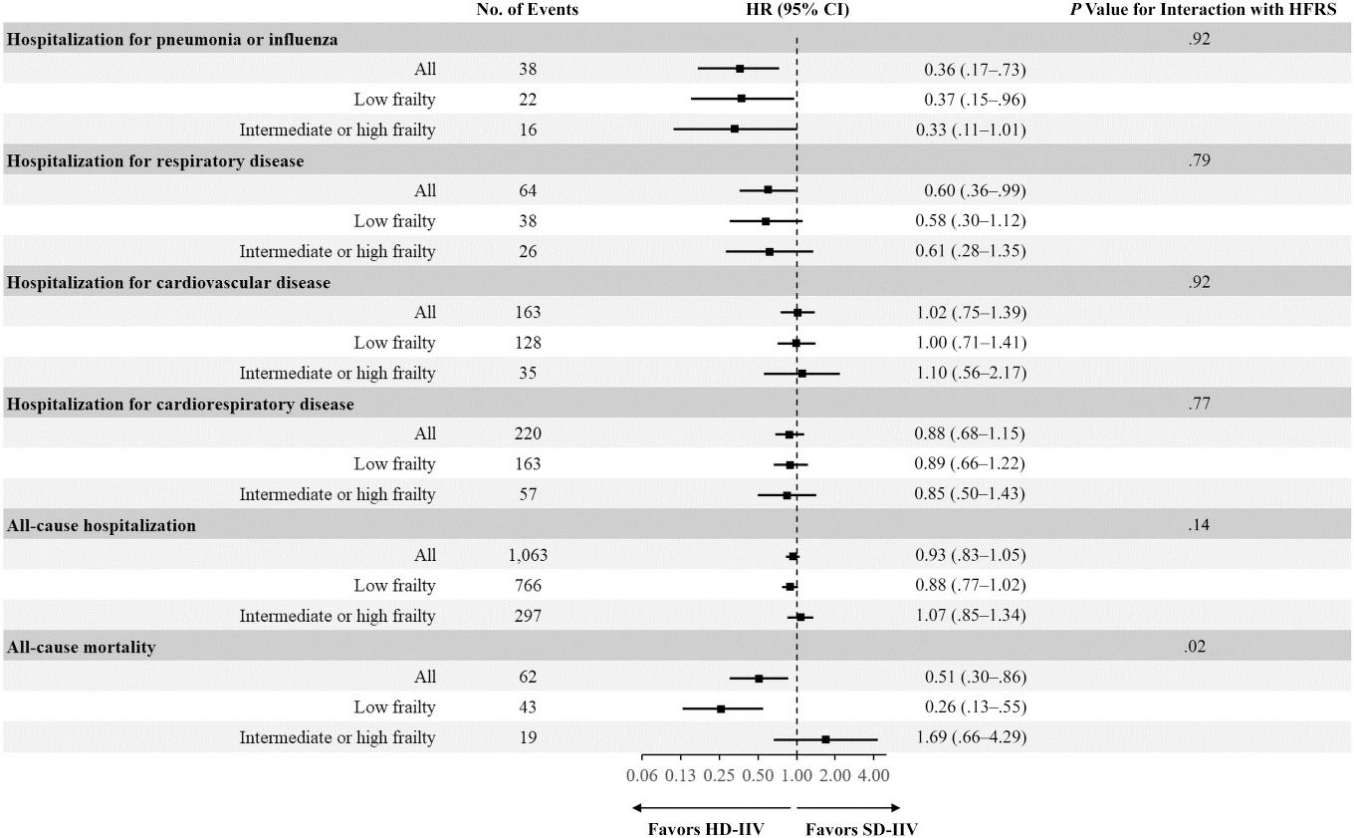
Relative effectiveness of high-dose inactivated influenza vaccination (HD-IIV) compared with standard-dose inactivated influenza vaccination (SD-IIV) against time to first events according to frailty status, defined according to the hospital frailty risk score (HFRS). Forest plot depicts hazard ratios (HRs) and 95% confidence intervals (CIs) for the individual outcomes according to HFRS. Low frailty corresponds to a HFRS of <5; intermediate or high frailty, to a HFRS of ≥5. The interaction with the HFRS was tested with HFRS as a continuous variable.

**Figure 2.**
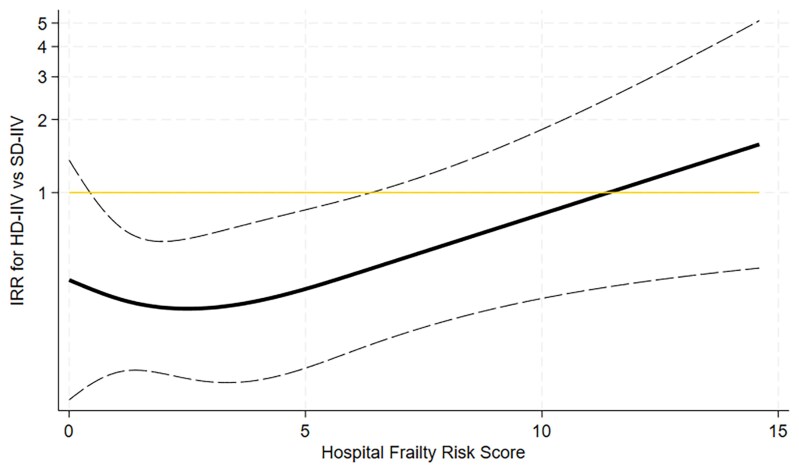
Association of high-dose influenza vaccination (HD-IIV) versus standard-dose influenza vaccination (SD-IIV) with all-cause mortality according to the hospital frailty risk score. Cubic spline curve represents incidence rate ratios (IRRs) (*solid black line*) and 95% confidence intervals (*dashed lines*), with SD-IIV as the control group (*horizontal line*).

In the time-to-first-event analysis, HD-IIV versus SD-IIV was associated with a lower hazard of hospitalizations for pneumonia or influenza, and this trend was consistent regardless of frailty status (HR, 0.37 [95% CI, .15–.96] in individuals with low frailty and 0.33 [.11–1.01] in those with intermediate or high frailty; *P* value for continuous interaction with HFRS [*P*_interaction_] = .92) (Figure 1). HD-IIV versus SD-IIV was also associated with a lower hazard of respiratory hospitalizations overall, and this association was consistent irrespective of the HFRS (HR, 0.58 [95% CI, .30–1.12] in individuals with low frailty and 0.61 [.28–1.35] in those with intermediate or high frailty; *P*_interaction_ = .79). HD-IIV was not associated with a significantly lower hazard of hospitalizations for cardiorespiratory or cardiovascular disease or any cause, regardless of frailty status (Figure 1). The HFRS significantly modified the association of HD-IIV versus SD-IIV with all-cause mortality (*P*_interaction_ = .02) (Figure 1). Among participants with low frailty, HD-IIV was associated with a lower hazard of all-cause mortality (HR, 0.26 [95% CI, .13–.55]), whereas no significant difference was observed among those with intermediate or high frailty (1.69 [.66–4.29]). Figure 2 displays the IRRs comparing the effects of HD-IIV versus SD-IIV on all-cause mortality according to the HFRS.

### Recurrent Events Analysis According to Frailty Status

**Figure 3.**
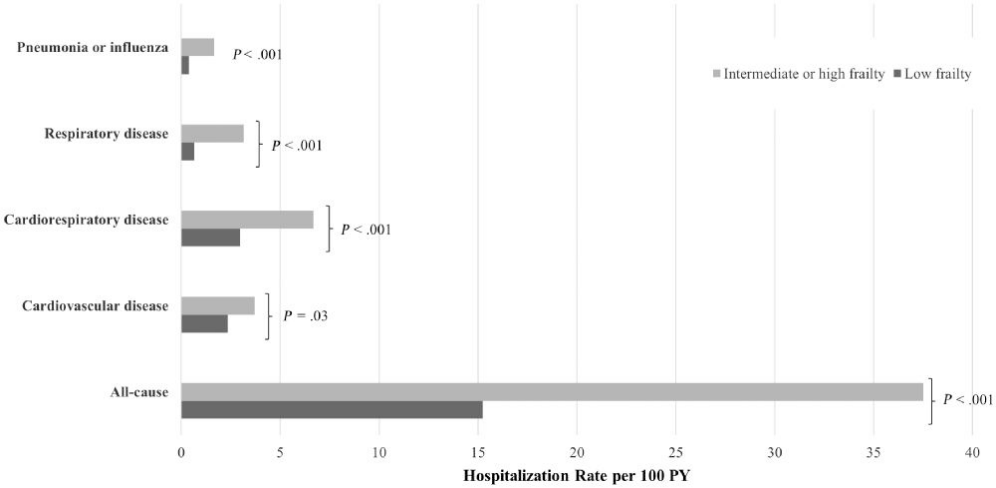
Hospitalization rate per 100 person-years (PY) according to frailty status, among those with low and intermediate or high frailty. Low frailty corresponds to a hospital frailty risk score (HFRS) of <5; intermediate or high frailty, to a HFRS of ≥5.

**Figure 4.**
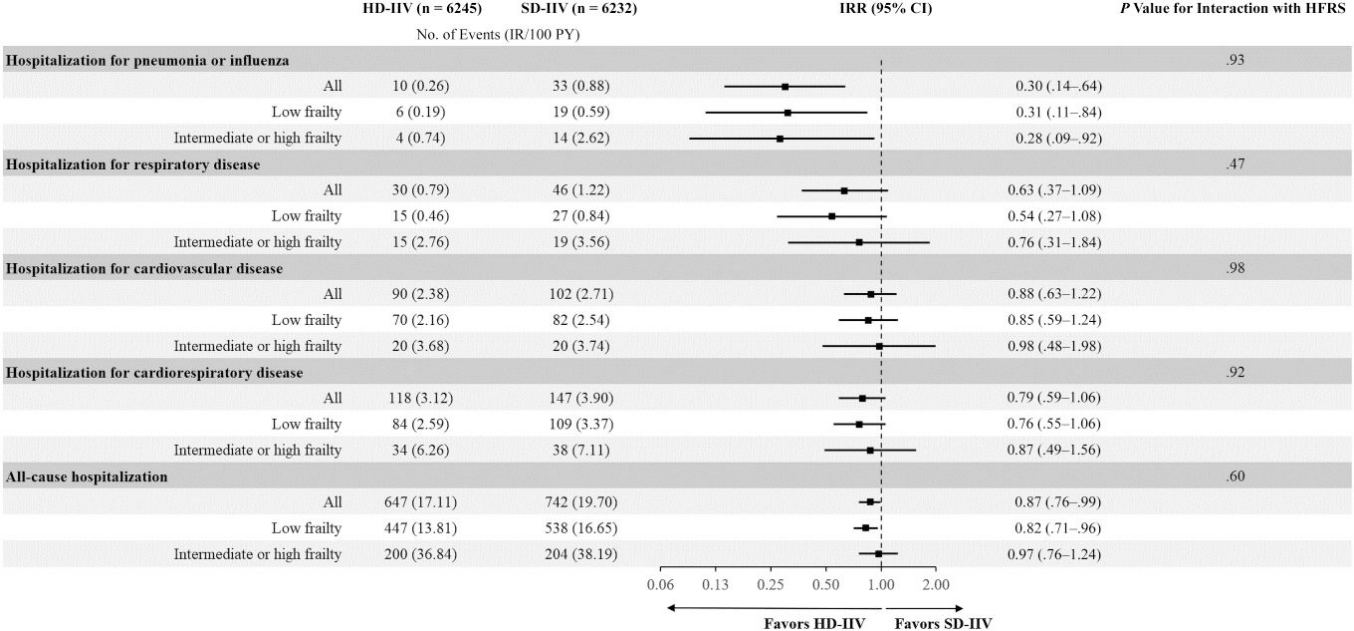
Relative effectiveness of high-dose influenza vaccination (HD-IIV) compared with standard-dose influenza vaccination (SD-IIV) against recurrent events according to frailty status. Forest plot depicts incidence rate (IR) ratios (IRRs) and 95% confidence intervals (CIs) as well IRs per 100 person-years (PY) for the individual outcomes according to frailty status. Low frailty corresponds to a hospital frailty risk score (HFRS) of <5; intermediate or high frailty, to a HFRS of ≥5. The interaction with the HFRS was tested with HFRS as a continuous variable.

The incidence rates of hospitalizations per 100 person-years for pneumonia or influenza and respiratory, cardiorespiratory, or cardiovascular disease as well as all-cause hospitalizations were higher in those with intermediate or high frailty than in those with low frailty (Figure 3). HD-IIV, compared with SD-IIV, was associated with a lower incidence rate of hospitalizations for pneumonia or influenza both in participants with low frailty (6 vs 19 events, respectively; IRR, 0.31 [95% CI, .11–.84]) and those with intermediate or high frailty (4 vs 14 events; IRR, 0.28 [.09–.92]; *P*_interaction_ = .93) (Figure 4). HD-IIV versus SD-IIV was also associated with a lower incidence rate of all-cause hospitalizations; this association was consistent irrespective of the HFRS (IRR, 0.82 [95% CI, .71–.96] in individuals with low frailty and 0.97 [.76–1.24] in those with intermediate or high frailty; *P*_interaction_ = .60). HD-IIV, compared with SD-IIV, was not associated with a significantly lower incidence rate of recurrent hospitalizations for respiratory, cardiorespiratory, or cardiovascular disease regardless of frailty status, although the outcomes trended in favor of HD-IIV (Figure 4). Results from an Andersen-Gill Cox model revealed similar estimates for the recurrent events and are shown in Supplementary Table 4.

### Increase in HFRS From Vaccination to End of Follow-up

On exclusion of those who died during follow-up (n = 62), the median (IQR) and mean (SD) HFRSs overall were 1.10 (0.00–3.30) and 2.27 (3.21), respectively, at baseline and 1.30 (0.00–3.60] and 2.46 (3.39), respectively, at follow-up (Supplementary Table 5). There was a significant increase in HFRS from the time of vaccination to the end of follow-up (*P* < .001 in paired analysis): the HFRS increased by a median (IQR) of 0.00 (0.00–0.00) and a mean (SD) of 0.19 (0.80) (n = 12 411).

In individuals randomized to HD-IIV, the median (IQR) and mean (SD) HFRSs at baseline were 1.10 (0.00–3.30) and 2.28 (3.21), respectively. Among individuals randomized to SD-IIV, these values were 1.10 (0.00, 3.30) and 2.26 (3.22), respectively (Supplementary Table 5). The HFRS increased significantly from the time of vaccination to the end of follow-up in both the HD-IIV and the SD-IIV group (*P* < .001 in paired analysis for both). The HFRSs at the end of follow-up did not differ significantly between participants receiving HD-IIV and those receiving SD-IIV (median [IQR], 1.30 [0.00–3.60] vs 1.30 [0.00–3.60], respectively [*P* = .75]; mean [SD], 2.45 [3.37] vs 2.46 [3.41] [*P* = .89]). Changes in HFRS did not differ significantly between those randomized to HD-IIV versus SD-IIV (median [IQR], 0.00 [0.00–0.00] vs 0.00 [0.00–0.00] [*P* = .07]; mean [SD], 0.17 [0.73] vs 0.20 [0.85)] [*P* = .052]) (Supplementary Table 5).

## DISCUSSION

In this post hoc analysis of a randomized, pragmatic feasibility trial, HD-IIV was associated with a lower risk of first and recurrent hospitalizations for pneumonia and influenza irrespective of frailty status, suggesting that HD-IIV may be similarly effective and beneficial for influenza-related outcomes in individuals with low and intermediate or high frailty. Moreover, HD-IIV versus SD-IIV was associated with lower incidence rates of all-cause hospitalizations regardless of frailty status, emphasizing the nonspecific beneficial effects of HD-IIV on downstream adverse outcomes in both patients with low and those with higher frailty risk. We did not find a significantly higher increase in HFRS from vaccination to the end of follow-up among individuals receiving SD-IIV compared with HD-IIV. Interestingly, the HFRS seemed to modify the association of HD-IIV versus SD-IIV with the all-cause mortality rate, suggesting a potential differential effect of HD-IIV by frailty status.

As frailty is a major risk factor for adverse outcomes after contracting influenza, with lower odds of recovery [29], more risk of significant functional decline [30], and worsening frailty, it is especially important to ensure high influenza vaccine efficacy and effectiveness among frail individuals. However, due to immunosenescence, older individuals exhibit lower immune responsiveness to vaccination [17]. The resulting decreased influenza vaccine efficacy may be particularly evident in older, frail individuals [7, 18], although the results regarding influenza vaccine–induced antibody titer in frail individuals are conflicting across studies [31–34]. One study found increased antibody titers after influenza vaccination in frail compared with nonfrail individuals, especially in those undergoing HD-IIV [35]. Moreover, HD-IIV, compared with SD-IIV, is associated with increased immune response and antibody titer regardless of frailty status [36, 37]. These results may therefore favor high HD-IIV among frail individuals.

In addition to improving vaccine efficacy, HD-IIV has also been shown to increase vaccine effectiveness by lowering the risk of cardiorespiratory events [14] and hospitalizations for pneumonia and influenza [23, 38, 39], compared with SD-IIV. Influenza vaccine effectiveness, however, has been shown to decrease with increasing level of frailty [19, 40]. Andrew et al [19] investigated trivalent influenza vaccine effectiveness against no vaccination in individuals aged ≥65 years hospitalized during the 2011–2012 influenza season. Frailty was assessed according to a validated frailty index based on a comprehensive geriatric assessment. Although the authors observed an overall moderate vaccine effectiveness in the entire population, that effectiveness decreased significantly with increasing level of frailty, further emphasizing the importance of considering frailty status in studies assessing influenza vaccine effectiveness [19]. The current study is the first to assess the effectiveness of HD-IIV compared with SD-IIV according to frailty status. As we did not observe evidence of a significant modification effect of the HFRS on HD-IIV versus SD-IIV for first and recurrent hospitalization for pneumonia or influenza, results were considered consistent across frailty groups. Accordingly, HD-IIV appeared similarly effective in preventing both first and recurrent hospitalizations for pneumonia and influenza in individuals with both low and higher frailty risk, suggesting similar benefit of HD-IIV in older patients irrespective of frailty status. Likewise, a cluster-randomized, single-blind trial found that trivalent HD-IIV versus SD-IIV decreased the risk of influenza-related hospitalizations in older nursing home residents, likely considered a high-risk frail group [39]. Regarding potential nonspecific effects of influenza vaccination, we also found a lower incidence rate of recurrent all-cause hospitalizations among individuals receiving HD-IIV versus SD-IIV, irrespective of frailty status. The indirect beneficial effects of HD-IIV compared with SD-IIV on all-cause hospitalization may therefore also be applicable regardless of frailty status.

A study assessing the effects of HD-IIV on disease severity among older adults in the United States over 3 influenza seasons from 2016 to 2019 found a numerically higher, but not statistically significant, reduction in the postinfluenza mortality rate in patients receiving HD-IIV versus SD-IIV [41]; however, this was based on observational data from US insurance claims over 3 influenza seasons with significant variability among individuals. DANFLU-1 demonstrated an association with significantly lower all-cause mortality risk among individuals receiving HD-IIV compared with SD-IIV [23]. Interestingly, however, in our post hoc analysis we observed that HD-IIV, compared with SD-IIV, was associated with a lower all-cause mortality risk among individuals with low frailty only. Although our findings should be considered exploratory and hypothesis generating, this could be explained by an inherently higher risk of adverse events and all-cause mortality among individuals with higher frailty. The increasing number of comorbid conditions associated with frailty may confer competing risks for all-cause mortality that may not be modifiable by influenza vaccination. As such, nonfrail individuals may derive a higher benefit of HD-IIV in lowering the all-cause mortality risk after influenza vaccination.

Our study does have limitations. As a post hoc analysis of a trial, not powered for clinical outcomes but rather designed to test feasibility, its results should be considered hypothesis generating and exploratory only. Moreover, no adjustments for multiple testing were done, which may increase the risk of a type 1 error. The associations should therefore be investigated further in adequately powered trials. For the purpose of this study, we used the HFRS to assess level of frailty based on *ICD-10* codes. Compared with other validated scores available based on clinical bedside assessments and tests [42, 43], the HFRS is based on passively collected administrative data. This may have led to some misclassification of frailty level and underestimation of frailty risk [44], particularly in relation to noninclusion of functional items that feature prominently in clinically based frailty measures. In addition, an increase in HFRS would reflect new accumulated *ICD-10* diagnoses, rather than increased severity of existing diagnoses or impacts on functional status (which are common, for example, after hospitalization including with influenza [45]); as such, changes in frailty identified using the HFRS may represent conservative estimates of a true change in frailty. It is also possible for frailty, assessed using clinically based measures, to improve [46]. Such improvement is less likely to be seen in the HFRS because it is based on diagnoses that are not usually cured—particularly when coded in administrative databases in which a past diagnosis sticks with the patient and is carried forward. However, the HFRS is a validated score to evaluate risk of frailty based on *ICD-10* codes [27], which has been used and validated in many different settings [47, 48], and it does allow estimation of frailty in large administrative databases and application pragmatic studies such as the current one. Finally, the HFRS was developed for adults >75 years of age and included *ICD-10* diagnoses within 2 years before assessment. In our study, we used the score in older adults 65–79 years of age and included diagnoses any time before vaccination in order to cover all relevant diagnoses for the included individuals. Inclusion of adults aged ≥80 years in future studies would provide more scope for comparison across higher levels of frailty.

In conclusion, in a post hoc analysis of a pragmatic, randomized feasibility trial, we found that HD-IIV was associated with a lower risk of first and recurrent hospitalizations for pneumonia or influenza irrespective of frailty level, suggesting a similar effectiveness of HD-IIV with influenza-related hospitalizations across frailty groups. However, the HFRS seemed to modify the association of HD-IIV versus SD-IIV with the all-cause mortality rate. These findings warrant further investigation in future, adequately powered trials.

## Supplementary Material

jiaf420_Supplementary_Data

## Supplementary Data

Supplementary materials are available at *The Journal of Infectious Diseases* online (http://jid.oxfordjournals.org/). Supplementary materials consist of data provided by the author that are published to benefit the reader. The posted materials are not copyedited. The contents of all supplementary data are the sole responsibility of the authors. Questions or messages regarding errors should be addressed to the author.
