# Supplementary material for: Relative Effectiveness of High-Dose Versus Standard-Dose Influenza Vaccination Against Hospitalizations and Deaths According to Frailty Score: A Post Hoc Analysis of the DANFLU-1 Randomized Trial
Source: J Infect Dis. 2025 Aug 13;233(1):e89–98. doi: 10.1093/infdis/jiaf420 (PMC12811850; doi:10.1093/infdis/jiaf420)
Supplement: jiaf420_Supplementary_Data [file jiaf420_supplementary_data.docx]

**Supplemental Material**

*”Relative Effectiveness of High-Dose versus Standard-Dose Influenza Vaccination Against Hospitalizations and Deaths According to Frailty Score: A Post Hoc Analysis of the DANFLU-1 Randomized Trial”*

**Table of contents**

[Supplemental Table S1. Overview of the calculation of the Hospital Frailty Risk Score and the prevalence of comorbidities in DANFLU-1 at baseline 2](#_Toc206502633)

[Supplemental Table S2. Baseline characteristics by randomization group in participants with low frailty risk (HFRS<5) (N=10,689) 15](#_Toc206502634)

[Supplemental Table S3. Baseline characteristics by randomization group in participants with intermediate and high frailty risk (HFRS≥5) (N=1,784) 17](#_Toc206502635)

[Supplemental Table S4. Recurrent events analysis using the Andersen-Gill Cox model 19](#_Toc206502636)

[Supplemental Table S5. Comparing frailty score at time of vaccination and end of follow-up 20](#_Toc206502637)

# **Supplemental Table S1.** Overview of the calculation of the Hospital Frailty Risk Score and the prevalence of comorbidities in DANFLU-1 at baseline

| **ICD-10 Code** | **ICD-10 Description** | **HD-IIV (n=6,243)** | | **SD-IIV (n=6,230)** | | **Total (n=12,473)** | |
| --- | --- | --- | --- | --- | --- | --- | --- |
| A04 | Other bacterial intestinal infections | 26 | 0,42% | 19 | 0,30% | 45 | 0,36% |
| A09 | Diarrhoea and gastroenteritis of presumed infectious origin | 58 | 0,93% | 52 | 0,83% | 110 | 0,88% |
| A41 | Other septicaemia | 120 | 1,92% | 120 | 1,93% | 240 | 1,92% |
| B95 | Streptococcus and staphylococcus as the cause of diseases classified to other chapters | 7 | 0,11% | 5 | 0,08% | 12 | 0,10% |
| B96 | Other bacterial agents as the cause of diseases classified to other chapters (secondary code) | 7 | 0,11% | <5 | - | - | - |
| D64 | Other anaemias | 69 | 1,10% | 76 | 1,22% | 145 | 1,16% |
| E05 | Thyrotoxicosis [hyperthyroidism] | 105 | 1,68% | 117 | 1,88% | 222 | 1,78% |
| E16 | Other disorders of pancreatic internal secretion | 19 | 0,30% | 10 | 0,16% | 29 | 0,23% |
| E53 | Deficiency of other B group vitamins | 5 | 0,08% | <5 | - | - | - |
| E55 | Vitamin D deficiency | 9 | 0,14% | 13 | 0,21% | 22 | 0,18% |
| E83 | Disorders of mineral metabolism | 48 | 0,77% | 35 | 0,56% | 83 | 0,67% |
| E86 | Volume depletion | 51 | 0,82% | 43 | 0,69% | 94 | 0,75% |
| E87 | Other disorders of fluid, electrolyte and acid- base balance | 107 | 1,71% | 96 | 1,54% | 203 | 1,63% |
| F00 | Dementia in Alzheimer's disease | 5 | 0,08% | 5 | 0,08% | 10 | 0,08% |
| F01 | Vascular dementia | <5 | - | <5 | - | - | - |
| F03 | Unspecified dementia | 7 | 0,11% | 8 | 0,13% | 15 | 0,12% |
| F05 | Delirium, not induced by alcohol and other psychoactive substances | 5 | 0,08% | 7 | 0,11% | 12 | 0,10% |
| F10 | Mental and behavioural disorders due to use of alcohol | 80 | 1,28% | 110 | 1,77% | 190 | 1,52% |
| F32 | Depressive episode | 22 | 0,35% | 35 | 0,56% | 57 | 0,46% |
| G20 | Parkinson's disease | 28 | 0,45% | 23 | 0,37% | 51 | 0,41% |
| G30 | Alzheimer's disease | 10 | 0,16% | <5 | - | - | - |
| G31 | Other degenerative diseases of nervous system, not elsewhere classified | <5 | - | 7 | 0,11% | - | - |
| G40 | Epilepsy | 60 | 0,96% | 67 | 1,08% | 127 | 1,02% |
| G45 | Transient cerebral ischaemic attacks and related syndromes | 200 | 3,20% | 192 | 3,08% | 392 | 3,14% |
| G81 | Hemiplegia | <5 | - | <5 | - | - | - |
| H54 | Blindness and low vision | 14 | 0,22% | 6 | 0,10% | 20 | 0,16% |
| H91 | Other hearing loss | 838 | 13,42% | 850 | 13,64% | 1688 | 13,53% |
| I63 | Cerebral Infarction | 165 | 2,64% | 163 | 2,62% | 328 | 2,63% |
| I67 | Other cerebrovascular diseases | 13 | 0,21% | 22 | 0,35% | 35 | 0,28% |
| I69 | Sequelae of cerebrovascular disease | 103 | 1,65% | 95 | 1,52% | 198 | 1,59% |
| I95 | Hypotension | 26 | 0,42% | 37 | 0,59% | 63 | 0,50% |
| J18 | Pneumonia, organism unspecified | 320 | 5,12% | 279 | 4,48% | 599 | 4,80% |
| J22 | Unspecified acute lower respiratory infection | 15 | 0,24% | 10 | 0,16% | 25 | 0,20% |
| J69 | Pneumonitis due to solids and liquids | <5 | - | 5 | 0,08% | - | - |
| J96 | Respiratory failure, not elsewhere classified | 65 | 1,04% | 64 | 1,03% | 129 | 1,03% |
| K26 | Duodenal ulcer | 23 | 0,37% | 15 | 0,24% | 38 | 0,30% |
| K52 | Other noninfective gastroenteritis and colitis | 96 | 1,54% | 106 | 1,70% | 202 | 1,62% |
| K59 | Other functional intestinal disorders | 209 | 3,35% | 252 | 4,04% | 461 | 3,69% |
| K92 | Other diseases of digestive system | 42 | 0,67% | 38 | 0,61% | 80 | 0,64% |
| L03 | Cellulitis | 23 | 0,37% | 29 | 0,47% | 52 | 0,42% |
| L08 | Other local infections of skin and subcutaneous tissue | 84 | 1,35% | 64 | 1,03% | 148 | 1,19% |
| L89 | Decubitus ulcer | 6 | 0,10% | 6 | 0,10% | 12 | 0,10% |
| L97 | Ulcer of lower limb, not elsewhere classified | 33 | 0,53% | 21 | 0,34% | 54 | 0,43% |
| M15 | Polyarthrosis | 55 | 0,88% | 48 | 0,77% | 103 | 0,83% |
| M19 | Other arthrosis | 410 | 6,57% | 389 | 6,24% | 799 | 6,40% |
| M25 | Other joint disorders, not elsewhere classified | 154 | 2,47% | 144 | 2,31% | 298 | 2,39% |
| M41 | Scoliosis | 20 | 0,32% | 19 | 0,30% | 39 | 0,31% |
| M48 | Spinal stenosis (secondary code only) | 241 | 3,86% | 249 | 4,00% | 490 | 3,93% |
| M79 | Other soft tissue disorders, not elsewhere classified | 352 | 5,64% | 361 | 5,79% | 713 | 5,71% |
| M80 | Osteoporosis with pathological fracture | 74 | 1,18% | 79 | 1,27% | 153 | 1,23% |
| M81 | Osteoporosis without pathological fracture | 384 | 6,15% | 339 | 5,44% | 723 | 5,79% |
| N17 | Acute renal failure | 26 | 0,42% | 22 | 0,35% | 48 | 0,38% |
| N18 | Chronic renal failure | 94 | 1,51% | 73 | 1,17% | 167 | 1,34% |
| N19 | Unspecified renal failure | 21 | 0,34% | 16 | 0,26% | 37 | 0,30% |
| N20 | Calculus of kidney and ureter | 182 | 2,91% | 205 | 3,29% | 387 | 3,10% |
| N28 | Other disorders of kidney and ureter, not elsewhere classified | 20 | 0,32% | 13 | 0,21% | 33 | 0,26% |
| N39 | Other disorders of urinary system (includes urinary tract infection and urinary incontinence) | 289 | 4,63% | 300 | 4,81% | 589 | 4,72% |
| R00 | Abnormalities of heart beat | 156 | 2,50% | 181 | 2,90% | 337 | 2,70% |
| R02 | Gangrene, not elsewhere classified | <5 | - | <5 | - | - | - |
| R11 | Nausea and vomiting | 52 | 0,83% | 47 | 0,75% | 99 | 0,79% |
| R13 | Dysphagia | 77 | 1,23% | 97 | 1,56% | 174 | 1,39% |
| R26 | Abnormalities of gait and mobility | 5 | 0,08% | 5 | 0,08% | 10 | 0,08% |
| R29 | Other symptoms and signs involving the nervous and musculoskeletal systems | 238 | 3,81% | 245 | 3,93% | 483 | 3,87% |
| R31 | Unspecified haematuria | 302 | 4,84% | 276 | 4,43% | 578 | 4,63% |
| R32 | Unspecified urinary incontinence | 73 | 1,17% | 72 | 1,16% | 145 | 1,16% |
| R33 | Retention of urine | 161 | 2,58% | 168 | 2,70% | 329 | 2,64% |
| R40 | Somnolence, stupor and coma | <5 | - | <5 | - | - | - |
| R41 | Other symptoms and signs involving cognitive functions and awareness | 50 | 0,80% | 36 | 0,58% | 86 | 0,69% |
| R44 | Other symptoms and signs involving general sensations and perceptions | <5 | - | <5 | - | - | - |
| R45 | Symptoms and signs involving emotional state | <5 | - | <5 | - | - | - |
| R47 | Speech disturbances, not elsewhere classified | <5 | - | <5 | - | - | - |
| R50 | Fever of unknown origin | 105 | 1,68% | 120 | 1,93% | 225 | 1,80% |
| R54 | Senility | 0 | 0,00% | 0 | 0,00% | 0 | 0,00% |
| R55 | Syncope and collapse | 263 | 4,21% | 246 | 3,95% | 509 | 4,08% |
| R56 | Convulsions, not elsewhere classified | 8 | 0,13% | 12 | 0,19% | 20 | 0,16% |
| R63 | Symptoms and signs concerning food and fluid intake | 37 | 0,59% | 44 | 0,71% | 81 | 0,65% |
| R69 | Unknown and unspecified causes of morbidity | <5 | - | <5 | - | - | - |
| R79 | Other abnormal findings of blood chemistry | 175 | 2,80% | 176 | 2,82% | 351 | 2,81% |
| R94 | Abnormal results of function studies | 9 | 0,14% | 15 | 0,24% | 24 | 0,19% |
| S00 | Superficial injury of head | 111 | 1,78% | 99 | 1,59% | 210 | 1,68% |
| S01 | Open wound of head | 317 | 5,08% | 332 | 5,33% | 649 | 5,20% |
| S06 | Intracranial injury | 95 | 1,52% | 89 | 1,43% | 184 | 1,47% |
| S09 | Other and unspecified injuries of head | 12 | 0,19% | <5 | - | - | - |
| S22 | Fracture of rib(s), sternum and thoracic spine | 72 | 1,15% | 65 | 1,04% | 137 | 1,10% |
| S32 | Fracture of lumbar spine and pelvis | 60 | 0,96% | 59 | 0,95% | 119 | 0,95% |
| S42 | Fracture of shoulder and upper arm | 227 | 3,63% | 209 | 3,35% | 436 | 3,49% |
| S51 | Open wound of forearm | 53 | 0,85% | 50 | 0,80% | 103 | 0,83% |
| S72 | Fracture of femur | 62 | 0,99% | 65 | 1,04% | 127 | 1,02% |
| S80 | Superficial injury of lower leg | 132 | 2,11% | 113 | 1,81% | 245 | 1,96% |
| T83 | Complications of genitourinary prosthetic devices, implants and grafts | 31 | 0,50% | 33 | 0,53% | 64 | 0,51% |
| U80 | Agent resistant to penicillin and related antibiotics | 0 | 0,00% | 0 | 0,00% | 0 | 0,00% |
| W01 | Fall on same level from slipping, tripping and stumbling | 0 | 0,00% | 0 | 0,00% | 0 | 0,00% |
| W06 | Fall involving bed | 0 | 0,00% | 0 | 0,00% | 0 | 0,00% |
| W10 | Fall on and from stairs and steps | 0 | 0,00% | 0 | 0,00% | 0 | 0,00% |
| W18 | Other fall on same level | 0 | 0,00% | 0 | 0,00% | 0 | 0,00% |
| W19 | Unspecified fall | 0 | 0,00% | 0 | 0,00% | 0 | 0,00% |
| X59 | Exposure to unspecified factor | 0 | 0,00% | 0 | 0,00% | 0 | 0,00% |
| Y84 | Other medical procedures as the cause of abnormal reaction of the patient | 0 | 0,00% | 0 | 0,00% | 0 | 0,00% |
| Y95 | Nosocomial condition | 0 | 0,00% | 0 | 0,00% | 0 | 0,00% |
| Z22 | Carrier of infectious disease | 0 | 0,00% | 0 | 0,00% | 0 | 0,00% |
| Z50 | Care involving use of rehabilitation procedures | 825 | 13,21% | 889 | 14,27% | 1714 | 13,74% |
| Z60 | Problems related to social environment | <5 | - | <5 | - | - | - |
| Z73 | Problems related to life-management difficulty | 7 | 0,11% | <5 | - | - | - |
| Z74 | Problems related to care-provider dependency | <5 | - | <5 | - | - | - |
| Z75 | Problems related to medical facilities and other health care | <5 | - | <5 | - | - | - |
| Z87 | Personal history of other diseases and conditions | 17 | 0,27% | 18 | 0,29% | 35 | 0,28% |
| Z91 | Personal history of risk-factors, not elsewhere classified | <5 | - | <5 | - | - | - |
| Z93 | Artificial opening status | 80 | 1,28% | 85 | 1,36% | 165 | 1,32% |
| Z99 | Dependence on enabling machines and devices | 25 | 0,40% | 27 | 0,43% | 52 | 0,42% |

# **Supplemental Table S2.** Baseline characteristics by randomization group in participants with low frailty risk (HFRS<5) (N=10,689)

|  | **IIV-HD**  **(N=5,343)** | **IIV-SD**  **(N=5,346)** |
| --- | --- | --- |
| Age, years; mean (SD) | 71.7 (3.9) | 71.5 (3.9) |
| Women, n (%) | 2,530 (47.4%) | 2,480 (46.4%) |
| Chronic lung disease, n (%) | 285 (5.3%) | 310 (5.8%) |
| Chronic obstructive pulmonary disease, n (%) | 133 (2.5%) | 134 (2.5%) |
| Chronic cardiovascular disease, n (%) | 880 (16.5%) | 966 (18.1%) |
| Ischemic heart disease, n (%) | 322 (6.0%) | 378 (7.1%) |
| Heart failure, n (%) | 91 (1.7%) | 93 (1.7%) |
| Atrial fibrillation, n (%) | 338 (6.3%) | 329 (6.2%) |
| Hypertension, n (%) | 2,656 (49.7%) | 2,629 (49.2%) |
| Diabetes, n (%) | 458 (8.6%) | 451 (8.4%) |
| Cerebrovascular disease, n (%) | 96 (1.8%) | 108 (2.0%) |
| Cancer, n (%) | 511 (9.6%) | 492 (9.2%) |
| Immunodeficiency, n (%) | 178 (3.3%) | 176 (3.3%) |
| FS, median (IQR) | 0.80 (0.00, 2.20) | 0.80 (0.00, 2.10) |

# **Supplemental Table S3.** Baseline characteristics by randomization group in participants with intermediate and high frailty risk (HFRS≥5) (N=1,784)

|  | **IIV-HD**  **(N=900)** | **IIV-SD**  **(N=884)** |
| --- | --- | --- |
| Age, years; mean (SD) | 72.6 (4.0) | 72.3 (4.0) |
| Women, n (%) | 426 (47.3%) | 440 (49.8%) |
| Chronic lung disease, n (%) | 150 (16.7%) | 105 (11.9%) |
| Chronic obstructive pulmonary disease, n (%) | 94 (10.4%) | 56 (6.3%) |
| Chronic cardiovascular disease, n (%) | 347 (38.6%) | 347 (39.3%) |
| Ischemic heart disease, n (%) | 128 (14.2%) | 134 (15.2%) |
| Heart failure, n (%) | 46 (5.1%) | 45 (5.1%) |
| Atrial fibrillation, n (%) | 120 (13.3%) | 91 (10.3%) |
| Hypertension, n (%) | 598 (66.4%) | 586 (66.3%) |
| Diabetes, n (%) | 116 (12.9%) | 137 (15.5%) |
| Cerebrovascular disease, n (%) | 123 (13.7%) | 129 (14.6%) |
| Cancer, n (%) | 184 (20.4%) | 176 (19.9%) |
| Immunodeficiency, n (%) | 66 (7.3%) | 63 (7.1%) |
| FS, median (IQR) | 7.40 (6.00, 9.80) | 7.00 (5.85, 10.00) |

# **Supplemental Table S4.** Recurrent events analysis using the Andersen-Gill Cox model

| **Endpoint/frailty status** | **HR (95% CI)** | **p for interaction with HFRS** |
| --- | --- | --- |
| **Hospitalization for pneumonia or influenza** |  | 0.90 |
| All participants (N=12,477) | 0.30 (0.14 to 0.64) |  |
| Low frailty (N=10,689) | 0.32 (0.12 to 0.85) |  |
| Intermediate or high frailty (N=1,784) | 0.28 (0.09 to 0.88) |  |
| **Hospitalization for respiratory disease** |  | 0.55 |
| All participants (N=12,477) | 0.65 (0.37 to 1.15) |  |
| Low frailty (N=10,689) | 0.56 (0.28 to 1.12) |  |
| Intermediate or high frailty (N=1,784) | 0.78 (0.32 to 1.88) |  |
| **Hospitalization for cardiovascular disease** |  | 0.999 |
| All participants (N=12,477) | 0.88 (0.63 to 1.23) |  |
| Low frailty (N=10,689) | 0.85 (0.59 to 1.24) |  |
| Intermediate or high frailty (N=1,784) | 0.98 (0.48 to 2.01) |  |
| **Hospitalization for cardiorespiratory disease** |  | 0.92 |
| All participants (N=12,477) | 0.80 (0.60 to 1.07) |  |
| Low frailty (N=10,689) | 0.77 (0.55 to 1.08) |  |
| Intermediate or high frailty (N=1,784) | 0.88 (0.49 to 1.57) |  |
| **All-cause hospitalization** |  | 0.27 |
| All participants (N=12,477) | 0.87 (0.76 to 0.99) |  |
| Low frailty (N=10,689) | 0.83 (0.71 to 0.97) |  |
| Intermediate or high frailty (N=1,784) | 0.96 (0.75 to 1.24) |  |

HR, hazard ratio; HFRS, hospital frailty risk score.

# **Supplemental Table S5.** Comparing frailty score at time of vaccination and end of follow-up

|  | **All (N=12,411*)** | **HD-IIV (N=6,222*)** | **SD-IIV (N=6,189*)** | **P** |
| --- | --- | --- | --- | --- |
| **Baseline** |  |  |  |  |
| FS, median (IQR) | 1.10 (0.00, 3.30) | 1.10 (0.00, 3.30) | 1.10 (0.00, 3.30) | 0.82 |
| FS, mean (SD) | 2.27 (3.21) | 2.28 (3.21) | 2.26 (3.22) | 0.74 |
| **Follow-up** |  |  |  |  |
| FS, median (IQR) | 1.30 (0.00, 3.60) | 1.30 (0.00, 3.60) | 1.30 (0.00, 3.60) | 0.75 |
| FS, mean (SD) | 2.46 (3.39) | 2.45 (3.37) | 2.46 (3.41) | 0.89 |
| **Change in FS** |  |  |  |  |
| ΔFS, median (IQR) | 0.00 (0.00, 0.00) | 0.00 (0.00, 0.00) | 0.00 (0.00, 0.00) | 0.070 |
| ΔFS, mean (SD) | 0.19 (0.80) | 0.17 (0.73) | 0.20 (0.85) | 0.052 |

*Excluding those who died during follow-up (N=62).
